# Supplementary material for: Spatiotemporal distribution and control of echinococcosis in Xinjiang, 2005–2023
Source: PLoS Negl Trop Dis. 2025 Dec 19;19(12):e0013775. doi: 10.1371/journal.pntd.0013775 (PMC12716753; doi:10.1371/journal.pntd.0013775)
Supplement: S1 Table — County IDs correspond to those used in all figures and tables. Data were obtained from the China Infectious Disease Surveillance and Reporting System (IDSR) and Xinjiang CDC records. (DOCX) [file pntd.0013775.s001.docx]

| **Supplementary Table S1. County id, names, and median reported incidence of echinococcosis in Xinjiang, 2005–2023. (per 100,000)** | | | | | | | | |
| --- | --- | --- | --- | --- | --- | --- | --- | --- |
| ID | Name | Median Reported Incidence | ID | Name | Median Reported Incidence | ID | Name | Median Reported Incidence |
| 0 | Luntai | 2.96 | 32 | Qira | 2.52 | 64 | Akto | 2.03 |
| 1 | Yuli | 1.51 | 33 | Yutian | 1.43 | 65 | Akqi | 13.08 |
| 2 | Ruoqiang | 3.61 | 34 | Minfeng | 5.63 | 66 | Xinyuan | 8.66 |
| 3 | Qiemo | 1.56 | 35 | Hotan | 0.66 | 67 | Zhaosu | 25.00 |
| 4 | Yanqi | 12.53 | 36 | Aksu | 1.55 | 68 | Tekes | 15.08 |
| 5 | Hejing | 19.83 | 37 | Kuqa | 2.79 | 69 | Nilka | 21.37 |
| 6 | Hexud | 15.39 | 38 | Wensu | 1.71 | 70 | Yining | 7.93 |
| 7 | Bohu | 12.67 | 39 | Shaya | 1.21 | 71 | Qapqal | 21.74 |
| 8 | Korla | 2.30 | 40 | Xinhe | 3.53 | 72 | Huocheng | 11.38 |
| 9 | Ulho | 10.02 | 41 | Baicheng | 2.49 | 73 | Gongliu | 13.22 |
| 10 | Baijiantan | 1.98 | 42 | Wushi | 2.46 | 74 | Bole | 12.71 |
| 11 | Karamay | 2.53 | 43 | Awat | 2.02 | 75 | Alashankou | 9.57 |
| 12 | Dushanzi | 2.79 | 44 | Kalpin | 12.45 | 76 | Igo | 10.09 |
| 13 | Changji | 6.81 | 45 | Tahcheng | 11.09 | 77 | Barkol | 9.03 |
| 14 | Fukang | 5.62 | 46 | Usu | 6.82 | 78 | Yizhou | 4.74 |
| 15 | Urumqi | 5.59 | 47 | Shawan | 3.44 | 79 | Jinghe | 11.40 |
| 16 | Midong | 3.81 | 48 | Toli | 26.19 | 80 | Wenquan | 22.13 |
| 17 | Dasaka | 8.38 | 49 | Yumin | 29.11 | 81 | Yengisar | 2.41 |
| 18 | Toutunhe | 3.88 | 50 | Hoboksar | 23.74 | 82 | Zepu | 0.92 |
| 19 | Terrazzo | 2.87 | 51 | Emin | 22.02 | 83 | Shache | 0.48 |
| 20 | Xinshi | 3.21 | 52 | Gaochang | 3.07 | 84 | Yecheng | 0.50 |
| 21 | Shaybak | 2.98 | 53 | Toksun | 9.71 | 85 | Markit | 0.91 |
| 22 | Tianshan | 2.50 | 54 | Shanshan | 4.16 | 86 | Yopurga | 1.56 |
| 23 | Hotan | 1.89 | 55 | Artux | 4.81 | 87 | Jiashi | 1.53 |
| 24 | Hutubi | 4.06 | 56 | Fuhai | 13.24 | 88 | Shufu | 1.60 |
| 25 | Manas | 4.21 | 57 | Fuyun | 8.62 | 89 | Shule | 1.48 |
| 26 | Qitai | 10.32 | 58 | Qinghe | 8.43 | 90 | Kashgar | 2.64 |
| 27 | Jimsar | 10.66 | 59 | Habahe | 14.14 | 91 | Kuytun | 1.58 |
| 28 | Mori | 28.11 | 60 | Jeminay | 23.27 | 92 | Khorgos | 4.18 |
| 29 | Moyu | 1.95 | 61 | Burqin | 10.27 | 93 | Yining | 7.20 |
| 30 | Pishan | 0.42 | 62 | Altai | 7.51 | 94 | Bachu | 1.45 |
| 31 | Lop | 0.77 | 63 | Wuqia | 22.20 | 95 | Tashkurgan | 8.47 |
