# Supplementary figures and images for: Spatiotemporal distribution and control of echinococcosis in Xinjiang, 2005–2023
Source: PLoS Negl Trop Dis. 2025 Dec 19;19(12):e0013775. doi: 10.1371/journal.pntd.0013775 (PMC12716753; doi:10.1371/journal.pntd.0013775)

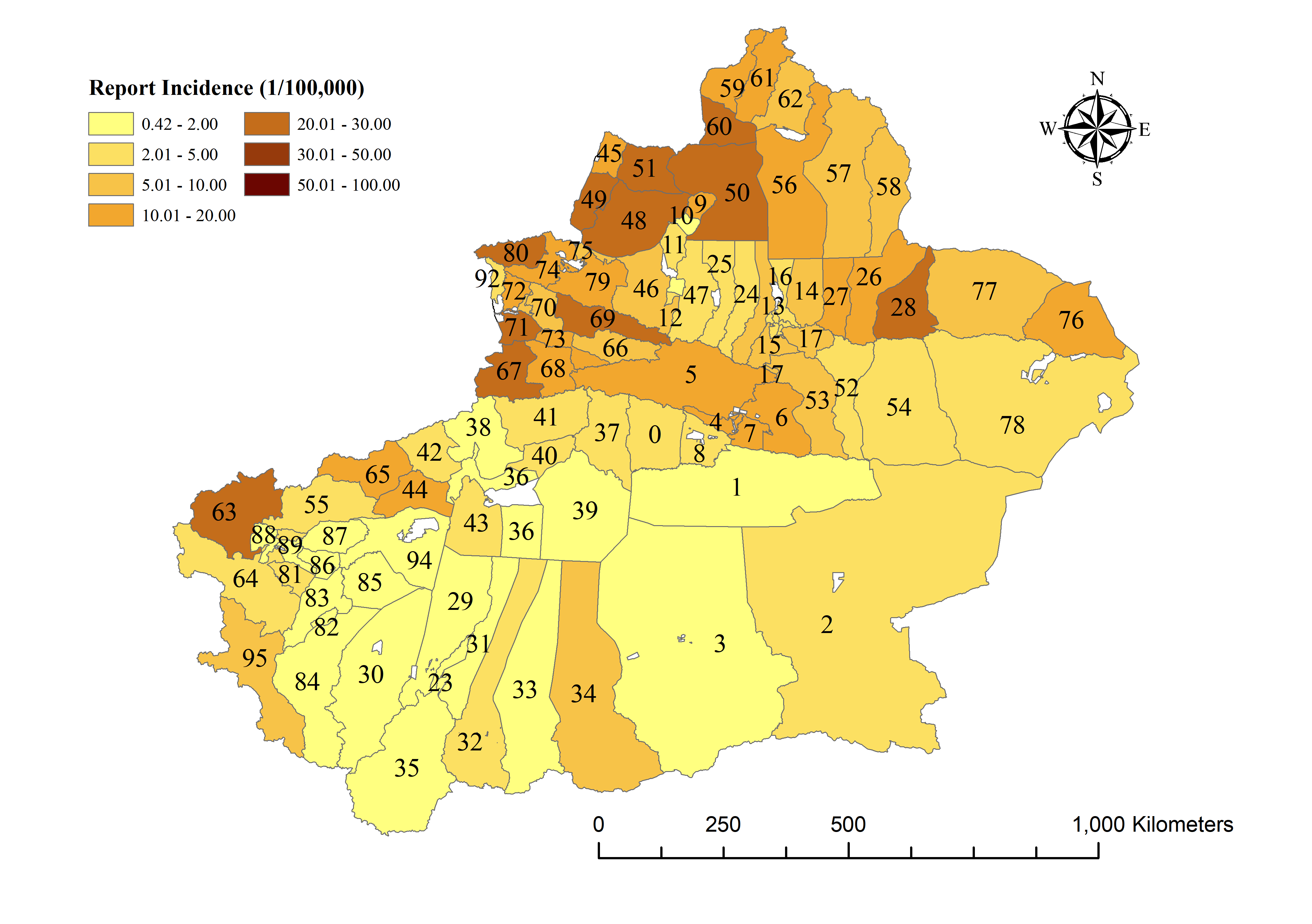

Supplement: S1 Fig — County IDs correspond to S1 Table. Data source: IDSR and Xinjiang CDC records. Base maps were sourced from the National Geographic Information Public Service Platform (www.tianditu.gov.cn, review number GS (2024) 0650) and are licensed for use under the Open Government Data License of China. Data were derived from the China Infectious Disease Surveillance and Reporting System (IDSR). (TIF) [file pntd.0013775.s002.tif]
