# Supplementary material for: Spatiotemporal distribution and control of echinococcosis in Xinjiang, 2005–2023
Source: PLoS Negl Trop Dis. 2025 Dec 19;19(12):e0013775. doi: 10.1371/journal.pntd.0013775 (PMC12716753; doi:10.1371/journal.pntd.0013775)
Supplement: Attachment — Submitted filename: Response to Reviewers.docx [file pntd.0013775.s004.docx]

PNTD-D-25-00532
Spatiotemporal Distribution and Control of Echinococcosis in Xinjiang, 2005-2023
PLOS Neglected Tropical Diseases

Reviewer #1 Wang Xu

Q1:

Use of the term "outbreak" (Line 194)

The authors describe a "severe initial outbreak" in Ulho County in 2005. However, echinococcosis is a chronic parasitic disease with insidious progression, which does not align with the classical epidemiological definition of an "outbreak" (typically acute and rapid transmission). Furthermore, since the study period starts in 2005, the term "initial" lacks context without pre-2005 data.

Recommendation : Replace "outbreak" with "high case detection" or similar phrasing. Provide historical context for temporal claims or clarify that "initial" refers to the study period rather than disease emergence.

**Response：**Thanks for your feedback. We have revised the sentence:

Lines:228-229

*“In 2005, Ulho's incidence rate was as high as 51.76/100,000, reflecting a high case detection.”*

Q2:

Interpretation of 2023 incidence increase (Lines 311–313)

The 28.30% year-on-year rise in 2023 is interpreted as a "clear upward trend," but this conclusion relies on a single data point without post-2023 validation. Such fluctuations could reflect surveillance artifacts (e.g., post-pandemic catch-up screenings) rather than true resurgence.

Recommendation: Acknowledge the limitation of interpreting short-term fluctuations and discuss alternative explanations (e.g., surveillance intensity).

**Response：**We thank the reviewer for this valuable suggestion. In response, we have removed the phrase “indicating a clear upward trend” and revised the text to adopt a more cautious interpretation, noting that further monitoring is needed to confirm whether the increase represents a sustained trend.

Lines:359-366

*“ Ai J. and colleagues, using a SARIMA model, predicted a slight increase in incidence between 2022 and 2025 [23]. Our study found that in 2023, the reported incidence rebounded to 3.99 per 100,000, representing a 28.3% year-on-year increase and returning to the 2020 level. This single-year increase should be interpreted with caution, as it may partly reflect surveillance artifacts rather than a true resurgence. Nonetheless, the finding underscores the need to sustain early screening, health education, and multi-level interventions in the post-pandemic period.”*

Q3:
Terminology: Echinococcus vs. echinococcosis (Figures/Tables)
The manuscript inconsistently uses Echinococcus (the parasite genus) where echinococcosis (the disease) is appropriate (e.g., in figures/tables). Additionally, Echinococcus should be italicized as a genus name.
Recommendation: Replace Echinococcus with "echinococcosis" or "Echinococcus infection" in all non-taxonomic contexts. Italicize Echinococcus when referring to the genus.

**Response：**Thanks for your feedback. We carefully reviewed all text, tables, and figures. Incorrect uses of Echinococcus have been replaced with “echinococcosis” or “Echinococcus infection” where appropriate. Echinococcus is now consistently italicized when referring to the genus.

Q4:

Units for subsidy funds (Line 299)
The currency unit for subsidies is unspecified.
Recommendation: State whether amounts are in CNY (RMB) or USD.
**Response：**Thank you for pointing out the issue. We have clarified that all subsidy amounts are in Chinese Yuan (CNY).

Lines:344-346

*“...promoting the construction of comprehensive intervention zones, and raising special subsidy standards (10,000 Chinese Yuan (CNY) for cystic echinococcosis and 20,000 CNY for alveolar echinococcosis).”*

Q5:

Redundancy in Table 5 and Figure 4

The spatial regression results are duplicated in Table5 and Figure4. Recommendation: Retain the table in the main text and move the figure to supplementary materials (or vice versa).

**Response：**Thank you for pointing out the potential redundancy between Table 5 and Figure 4, and we appreciate your attention to how the research results are presented. To clarify the content and relationship of these two materials, the specific explanation is as follows:

Table 5: Presents the results of spatial analysis on echinococcosis in Xinjiang from 2005 to 2023 in a data table format, including global autocorrelation (Moran'I value, etc.), local autocorrelation (number of High-High/Low-Low clusters), and coldspot-hotspot analysis (number of hotspots/coldspots). It only shows "quantitative data" without specific location information.

Figure 4: Uses a map visualization format, marking the specific county-level locations of echinococcosis hotspots and coldspots in each year with colors/symbols. It only shows "spatial distribution" without statistical data.

Relationship between the two: They share the same data source and are complementary in "quantitative statistics-spatial visualization". Table 5 indicates "how many" (quantities), while Figure 4 indicates "where" (locations). Together, they form a complete spatial analysis logic and are not simple repetitions.

Q6:

Map visualization improvements (Figures 3 and 4)

County labels are overcrowded, hindering readability.

Recommendation: Use numbered codes for counties, with a legend highlighting high/low-incidence areas (e.g., hotspots in red).

**Response：**Thank you for your suggestion on improving the map visualization of Figures 3 and 4.

To address the overcrowded county labels that hindered readability, we have adjusted both figures by replacing direct county name labels with numbered codes. Furthermore, we have supplemented Appendix S1, which includes the county ID, county names, and median reported incidence of echinococcosis in Xinjiang from 2005 to 2023.

Regarding the legend and color scheme: For Figure 4 (the coldspot-hotspot analysis map), we retain the existing blue/red color coding to clearly identify disease hotspots (red) and coldspots (red), which aligns with the map’s purpose of distinguishing high- and low-incidence areas. For Figure 3 (the spatial distribution thematic map), since it adopts a 7-category classification based on actual incidence values—this classification does not directly define "high-incidence" or "low-incidence" areas, and using multiple distinct colors would cause visual clutter—we continue to use a single color gradient for display to ensure clarity.

Q7:

Year selection rationale in the Maps (Figures 3 and 4)
The rationale for selecting specific years (e.g., 2005, 2010, 2015, 2020) is unclear.
Recommendation: Justify year selection (e.g., alignment with policy changes, surveillance milestones).

**Response：**Thank you for your comment on the year selection rationale in Figures 3 and 4. Our study covers the data period 2005–2023, and we have generated spatial distribution maps for every single year. To balance the presentation of key milestones and page aesthetics (avoiding information redundancy), we selected 8 representative years (2006, 2007, 2010, 2017, 2019, 2020, 2022, 2023) based on the following considerations:

（1）Data completeness: The 2005 dataset had 27 missing values, and no valid hotspots/coldspots were identified in the coldspot-hotspot analysis. By contrast, 2006 had more complete data, making it a more suitable starting year for interannual comparisons.

（2）Policy alignment: Echinococcosis was designated as a major infectious disease eligible for free treatment in 2006; 2007 was selected to reflect the early implementation phase of this policy.

（3）Epidemiological milestones: 2017 was identified as the peak year of incidence via the joinpoint model, representing a critical turning point in the epidemiological trend. Additionally, a universal health check-up program was launched in Xinjiang in September 2016.

（4）Key event benchmarks: 2019 was chosen as the year before the COVID-19 pandemic, 2020 as the pandemic period, and 2023 as the post-pandemic year—these years enable analysis of pandemic impacts on disease distribution. Furthermore, in 2019, all 81 echinococcosis-endemic counties had fully launched surveillance work.

（5）Temporal balance: 2010was selected as a mid-term year (between 2006 and 2017) to capture intermediate changes in the long-term trend.dditionally, the Action Plan for Echinococcosis Control (2010–2015) was issued in 2010.

These timestamps have also been reflected in the Discussion section.

Lines325-366

*“Since December 2004, echinococcosis has been included in the management of Category C notifiable infectious diseases under the Infectious Disease Prevention and Control Law, and in 2005 the national control program was launched, with central government funds transferred to support control activities in Xinjiang [13-14]. In 2006, echinococcosis was designated as a major infectious disease eligible for free treatment. In 2010, the “Action Plan for Echinococcosis Control (2010–2015)” was implemented, with core measures including dog registration and regular deworming, slaughterhouse inspection and safe disposal, targeted population screening and treatment, improvement of drinking water in pastoral areas, and multi-level health education [15-17]. Since 2016, Xinjiang has launched a universal health check-up program, offering annual free examinations for registered residents, which substantially improved the capacity for early case detection [18]. Therefore, the continued increase in reported incidence from 2005 to 2017 reflects not only the cumulative effect of ongoing transmission but also a significant rise in case detection driven by policy initiatives and active screening.*

*From 2017 to 2023, the reported incidence of echinococcosis in Xinjiang declined significantly, with an APC of −17.71% (95% CI: −27.81 to −10.48), indicating the positive impact of control measures [19]. During this period, the government continued to increase financial and technical input, achieving routine surveillance in all 81 endemic counties by 2019, promoting the construction of comprehensive intervention zones, and raising special subsidy standards (10,000 Chinese Yuan (CNY) for cystic echinococcosis and 20,000 CNY for alveolar echinococcosis). In addition, extensive health education campaigns were implemented, effectively improving residents’ awareness and knowledge of prevention and control [20-21]. By 2019, prior to the COVID-19 pandemic, the reported incidence had fallen to 6.89 per 100,000, representing a 36.6% decrease from the 2017 peak. However, during the COVID-19 outbreak from 2020 to 2022, substantial prevention and control resources were redirected to pandemic response, limiting human and material inputs for echinococcosis control. Routine surveillance and intervention schedules were disrupted, with reduced monitoring frequency and delayed implementation of control measures in some areas, thereby weakening the effectiveness of interventions [22]. In this period, the reported incidence was 4.04, 3.65, and 3.11 per 100,000, respectively, showing a continued decline, though the decrease may partly reflect underreporting due to limited surveillance during the pandemic. In the post-pandemic era, close attention should be paid to the potential rebound of incidence. Ai J. and colleagues, using a SARIMA model, predicted a slight increase in incidence between 2022 and 2025 [23]. Our study found that in 2023, the reported incidence rebounded to 3.99 per 100,000, representing a 28.3% year-on-year increase and returning to the 2020 level. This single-year increase should be interpreted with caution, as it may partly reflect surveillance artifacts rather than a true resurgence. Nonetheless, the finding underscores the need to sustain early screening, health education, and multi-level interventions in the post-pandemic period.”*

Q8：

Supplementary spatial analysis

Cumulative annual incidence or average annual incidence maps could better illustrate long-term spatiotemporal patterns. Recommendation: Add such maps as supplementary figures.

**Response：**Thank you for your suggestion on supplementary spatial analysis. Due to data limitations—our incidence data comes from the National Notifiable Infectious Diseases Surveillance System, which only provides annual county-level incidence results (no annual population data)—we cannot calculate cumulative or average annual incidence.

We have already mapped 8 representative years (2006, 2007, 2010, 2017, 2019, 2020, 2022, 2023) to visually show long-term spatial variation in incidence. To further illustrate long-term patterns, we will add an "annual incidence median map" (calculated using annual incidence data from 2005–2023) as supplementary material, which reflects long-term average incidence levels without relying on population data.

**Supplementary Figure S1. Median Reported Incidence of Echinococcosis in Xinjiang by County (2005–2023)**

Q9：

Additional evaluation opinion of "incidence rate" terminology
The manuscript uses "incidence rate" throughout, but echinococcosis is a chronic condition where detected cases often reflect long-term infections rather than new acute infections. As noted in the Discussion, case identification may correlate with screening programs rather than true incidence. Recommendation: Clarify in the Methods/Discussion that "incidence" here refers to reported cases (not true disease onset) and discuss screening-driven biases.

**Response：**Thank you for your comment on the "incidence rate" terminology. All "incidence rate" data in this study were directly obtained from China’s National Infectious Disease Surveillance and Reporting System (IDSR), and we acknowledge the need for caution when using this term for the chronic echinococcosis. In response to your suggestion, we have revised the manuscript: in the Methods section, we clarified the data source of "incidence rate" and specified its operational definition as "given the chronic nature of echinococcosis, the term 'incidence rate' in this context should be more accurately interpreted as the reported case detection rate, based on both passive surveillance and active screening"; in the Discussion section, we also addressed potential interpretation biases, emphasizing that the observed variations in "incidence rate" may not only reflect true epidemiological trends but could also be influenced by regional differences in screening intensity, diagnostic capacity, and reporting completeness.

Lines:99-110

*“Data on cases of echinococcosis in Xinjiang Uygur Autonomous Region and its 96 districts/counties during 2005–2023 were obtained from the China Infectious Disease Surveillance and Reporting System (IDSR), a national platform for mandatory infectious disease reporting. Cases were diagnosed according to the National Diagnostic Standard for Echinococcosis (WS 257-2006), which considers epidemiological history, clinical symptoms, imaging findings, and laboratory results. All reported cases were reviewed through multi-level verification at county, prefecture, regional, and national Centers for Disease Control and Prevention (CDC). The incidence data were directly obtained from IDSR and were not independently calculated by the authors. Because echinococcosis is a chronic disease, the reported incidence reflects the number of confirmed and reported cases within a given period rather than the true rate of new infections. ”*

*“It should be noted that the reported incidence reflects only diagnosed cases registered in the surveillance system, rather than the actual rate of new infections. Given the chronic course of echinococcosis, the reported incidence is highly influenced by the extent of surveillance coverage and the intensity of active case detection.”*

**Reviewer #2:**

Q1:

Spatiotemporal models are powerful tools especially for analyzing and predicting the trend of echinococcosis given that its transmission is so complex. However, the data factors in this study are so simple including human cases in different areas and different time points. The simple size was OK for the analysis. The authors should provide details of control measures.

**Response：**Thank you for your valuable comments. We fully agree that clarifying details of control measures is critical for understanding the transmission dynamics of echinococcosis. The main objective of our study was to provide the first long-term spatiotemporal characterization of echinococcosis in Xinjiang from 2005 to 2025 and to project future trends, based on standardized surveillance data covering all 96 counties. This approach ensured data comparability but limited our analysis to reported incidence. We also acknowledge that, due to data constraints, local variations in dog management, health education, and livestock interventions could not be systematically incorporated.

In response to your suggestions, we have made the following revisions in the manuscript:

Background: We added a concise paragraph systematically reviewing the major national and Xinjiang-specific control programs since 2005 and their core measures, with relevant references, to provide context on the long-term prevention and control efforts.

Figure presentation: In Figure 1, we added a timeline overlaying the initiation or expansion of major control programs with the annual incidence curve, allowing readers to intuitively understand the relationship between policy measures and fluctuations in incidence.

Discussion: We elaborated on the dynamic changes in reported incidence in relation to policy implementation, highlighting the association between interventions and epidemiological trends, while also noting other possible influencing factors such as surveillance coverage, active screening, and reporting bias.

Limitations: We stated that the lack of quantitative county-level intervention data limited our ability to systematically evaluate the impact of different intervention intensities on incidence. We further suggested that future research could conduct county-level comparative studies (e.g., counties with active screening versus those without) to more accurately assess intervention effects.

These revisions aim to present more clearly the relationship between control measures and changes in reported incidence, while also clarifying the limitations of our study and potential directions for future research. We believe these adjustments will help readers gain a more comprehensive understanding of the long-term epidemiological trends of echinococcosis and the effectiveness of control strategies in Xinjiang.

*“To address this high prevalence, the Chinese government and Xinjiang regional authorities have implemented a series of echinococcosis control programs since 2005. These measures include free treatment for patients, regular dog deworming campaigns, targeted population screening, health education initiatives, and improvements in pastoral sanitation. Such programs have been crucial in enhancing case detection and reducing transmission in endemic areas [8-9].”*

Lines325-366

*“Since December 2004, echinococcosis has been included in the management of Category C notifiable infectious diseases under the Infectious Disease Prevention and Control Law, and in 2005 the national control program was launched, with central government funds transferred to support control activities in Xinjiang [13-14]. In 2006, echinococcosis was designated as a major infectious disease eligible for free treatment. In 2010, the “Action Plan for Echinococcosis Control (2010–2015)” was implemented, with core measures including dog registration and regular deworming, slaughterhouse inspection and safe disposal, targeted population screening and treatment, improvement of drinking water in pastoral areas, and multi-level health education [15-17]. Since 2016, Xinjiang has launched a universal health check-up program, offering annual free examinations for registered residents, which substantially improved the capacity for early case detection [18]. Therefore, the continued increase in reported incidence from 2005 to 2017 reflects not only the cumulative effect of ongoing transmission but also a significant rise in case detection driven by policy initiatives and active screening.*

*From 2017 to 2023, the reported incidence of echinococcosis in Xinjiang declined significantly, with an APC of −17.71% (95% CI: −27.81 to −10.48), indicating the positive impact of control measures [19]. During this period, the government continued to increase financial and technical input, achieving routine surveillance in all 81 endemic counties by 2019, promoting the construction of comprehensive intervention zones, and raising special subsidy standards (10,000 Chinese Yuan (CNY) for cystic echinococcosis and 20,000 CNY for alveolar echinococcosis). In addition, extensive health education campaigns were implemented, effectively improving residents’ awareness and knowledge of prevention and control [20-21]. By 2019, prior to the COVID-19 pandemic, the reported incidence had fallen to 6.89 per 100,000, representing a 36.6% decrease from the 2017 peak. However, during the COVID-19 outbreak from 2020 to 2022, substantial prevention and control resources were redirected to pandemic response, limiting human and material inputs for echinococcosis control. Routine surveillance and intervention schedules were disrupted, with reduced monitoring frequency and delayed implementation of control measures in some areas, thereby weakening the effectiveness of interventions [22]. In this period, the reported incidence was 4.04, 3.65, and 3.11 per 100,000, respectively, showing a continued decline, though the decrease may partly reflect underreporting due to limited surveillance during the pandemic. In the post-pandemic era, close attention should be paid to the potential rebound of incidence. Ai J. and colleagues, using a SARIMA model, predicted a slight increase in incidence between 2022 and 2025 [23]. Our study found that in 2023, the reported incidence rebounded to 3.99 per 100,000, representing a 28.3% year-on-year increase and returning to the 2020 level. This single-year increase should be interpreted with caution, as it may partly reflect surveillance artifacts rather than a true resurgence. Nonetheless, the finding underscores the need to sustain early screening, health education, and multi-level interventions in the post-pandemic period.”*

Lines: 410-426

*“This study has several limitations. First, the early monitoring system in Xinjiang was incomplete, resulting in missing incidence data for certain years and counties, which affects the continuity of trend analysis and the completeness of spatial distribution research. Second, the reported incidence data reflect only diagnosed and registered cases, which may differ from the true infection rate due to underreporting or variations in surveillance coverage and active case-finding intensity. Third, the study lacks quantitative data on the implementation of control interventions (e.g., dog deworming, livestock vaccination, and active screening campaigns), which limits the ability to directly evaluate the impact of these measures on incidence trends. Fourth, this study mainly considers temporal and spatial factors, without incorporating climate change, socio-economic factors, and other potential determinants, which may limit a comprehensive understanding of the drivers of incidence changes.*

*Given these limitations, future research could incorporate climate and socio-economic variables, obtain quantitative intervention data [30], and conduct county-level comparative studies (e.g., active screening vs. non-active screening areas) to more systematically assess the effectiveness of control measures and further elucidate the epidemiological patterns of echinococcosis in Xinjiang.”*

Q2:

In this study entitled “Spatiotemporal Distribution and Control of Echinococcosis in Xinjiang, 2005-2023,” Zhe Yin et al. collected annual human echinococcosis cases from each county of Xinjiang Uygur Autonomous Region (province level) to fit the spatiotemporal model, showing the distribution of echinococcosis in both spatial and temporal dimensions. They also used the annual case reports from each county to determine the control progress from 2005 to 2023. Given that echinococcosis is an important zoonosis and very few control study in recent years, the data is very crucial if the authors give more details on the control measures used in the control programme. Further more, in this study, the authors showed that after the control program was implemented, the number of human cases increased, peaking in 2017 at 10.8/1,000,000. The authors suggested that this might be due to the active population screening procedure. Additionally, they mentioned that the control program was not implemented evenly or equally in these counties in Xinjiang from 2005 to 2017. I believe these data are useful for demonstrating that active population screening may cause an increase in reported human cases. Therefore, the authors may design a study comparing these counties with active screening to those without screening. It is not appropriate to mix these data together to show an “increasing” or “decreasing” trend. After 2017, all the targeted counties were implementing the control program, the authors should provide details about the control measures used and how the program was monitored. Furthermore, if they claim that the incidence from the active screening procedure can be used to profile the control program, they should provide more details about the population screening, such as whether it was conducted on the entire population.

**Response：**We sincerely thank you for this constructive comment. We fully agree that detailed information on control measures and active population screening is crucial for interpreting temporal trends in reported echinococcosis incidence.

In response to your suggestion, we have made the following revisions in the manuscript:

A concise paragraph was added in the Background to systematically summarize the main national and Xinjiang-level control programs since 2005 and their core measures, with relevant references included.

A timeline was added to Figure 1, overlaying the initiation or expansion of major control programs onto the annual reported incidence curve, allowing readers to intuitively understand the relationship between policy measures and incidence fluctuations.

In the Discussion, we elaborated on how the reported incidence dynamically changed in response to policy implementation, clarifying the intrinsic link between control measures and incidence trends.

In the Limitations section, we noted that, due to the lack of quantitative county-level intervention data, it was not possible to systematically analyze the specific impact of different intervention intensities on reported incidence. We also suggested that future studies could conduct comparative analyses (e.g., counties with active screening vs. counties without) to more precisely evaluate intervention effects.

These revisions aim to more clearly present the relationship between control measures and changes in reported incidence, while highlighting the study’s limitations and directions for future research.

Spatiotemporal models are good and powerful models to analyze the spread of infectious diseases. In terms of zoonosis, the data should not only include the time and location of cases and incidence rates, but also factors such as health care capacity, livestock husbandry, agricultural activities, rural populations, and education levels, which are likely to play significant roles in determining the prevalence of CE. Most importantly, the model should be used to describe the transmission pathways of this helminth zoonosis across different regions over time, to identify high-risk areas and periods, and most importantly, to forecast potential future trends of the epidemic to support public health and design control measures.

Response：We thank the reviewer for this valuable comment. We fully agree that details of control measures and local interventions are critical for understanding the transmission dynamics of echinococcosis. The main aim of our study was to provide the first long-term spatiotemporal characterization of echinococcosis across Xinjiang from 2005 to 2023, using standardized surveillance data covering all 96 counties, and to project potential future trends. This approach ensured comparability across time and space but limited our analysis to reported incidence.

We acknowledge that local variations in dog management, health education, livestock husbandry, and other socio-economic factors could not be systematically incorporated due to data unavailability. In addition, quantitative data on the intensity and coverage of interventions were not accessible, which we now explicitly state as a limitation. Future studies could integrate such intervention and contextual data into causal or mechanistic models to better describe transmission pathways, identify high-risk areas and periods, and forecast epidemic trends, thereby providing more actionable guidance for public health planning and control strategies.

Lines: 410-426

*“This study has several limitations. First, the early monitoring system in Xinjiang was incomplete, resulting in missing incidence data for certain years and counties, which affects the continuity of trend analysis and the completeness of spatial distribution research. Second, the reported incidence data reflect only diagnosed and registered cases, which may differ from the true infection rate due to underreporting or variations in surveillance coverage and active case-finding intensity. Third, the study lacks quantitative data on the implementation of control interventions (e.g., dog deworming, livestock vaccination, and active screening campaigns), which limits the ability to directly evaluate the impact of these measures on incidence trends. Fourth, this study mainly considers temporal and spatial factors, without incorporating climate change, socio-economic factors, and other potential determinants, which may limit a comprehensive understanding of the drivers of incidence changes.*

*Given these limitations, future research could incorporate climate and socio-economic variables, obtain quantitative intervention data [30], and conduct county-level comparative studies (e.g., active screening vs. non-active screening areas) to more systematically assess the effectiveness of control measures and further elucidate the epidemiological patterns of echinococcosis in Xinjiang.”*

Q3:

Abstract results: “The reported incidence of echinococcosis in Xinjiang ranged from 1.95 to 10.85 per 100,000.” In which years did these figures occur? Does this mean the incidence increased after the control program was implemented?

**Response：**Thank you for your comment. In the revised abstract, the reported incidence of echinococcosis ranged from 1.95 per 100,000 in 2005 to 10.85 per 100,000 in 2017. The subsequent increase in reported incidence after 2005 does not indicate that the control program was ineffective. Rather, it reflects multiple factors including expanded surveillance coverage, increased active case-finding, and improved case detection capacity. We have revised the Discussion section to provide a detailed description of the dynamic changes in reported incidence in relation to the implementation of relevant policies.

Q4:

Abstract: The authors should present some specific data in the abstract results, rather than just using terms like “low,” “hotspot,” or “cold spots.”

**Response：**We appreciate the suggestion. Specific data have now been added to the abstract results, including annual incidence rates, the number and distribution of hotspot and cold spot counties, to provide clearer and more concrete information.

Q5:

What does “spatial lag and temporal lag significantly affecting incidence rates” mean? Please provide data to support these results!

**Response：**Regarding the statement that "spatial lag and temporal lag significantly affected incidence rates," the STAR model results provide quantitative support, including regression coefficients (β), significance levels (P-values), residual ranges, and model fit indicators (AIC). These data are now presented in the results section to support the conclusion that year, spatial lag, and temporal lag significantly influence incidence rates.

Q6:

Abstract Conclusion: The conclusion should clearly summarize the epidemiological situation and control efficacy, aligning with the title and aims of the study.

**Response：**Thank you for your valuable comment. We have revised the abstract conclusion to clearly summarize both the spatiotemporal epidemiological situation of echinococcosis in Xinjiang and the impact of national and regional control programs. The revised conclusion emphasizes the observed heterogeneity, the reduction in incidence after 2017, and the continued need for sustained and regionally coordinated prevention and control strategies, thereby aligning more closely with the title and aims of the study.

Lines:12-38

***“Objective:*** *This study investigated the epidemiological characteristics of echinococcosis in Xinjiang, aiming to provide evidence for evaluating prevention and control progress and guiding future strategies.* ***Methods:*** *Reported echinococcosis incidence data from Xinjiang during 2005–2023 were obtained from the China Infectious Disease Surveillance and Reporting System. Temporal trends and spatial distributionwere analyzed using joinpoint regression and spatial autocorrelation methods. Additionally, a linear regression model (LR), spatial autoregressive model (SAR), and spatiotemporal autoregressive model (STAR) were constructed to quantitatively assess the impact of factors such as year, spatial lag, and temporal lag on incidence rates.* ***Results*** *: The reported incidence ranged from 1.95 per 100,000 in 2005 to 10.85 per 100,000 in 2017, followed by a gradual decline, though levels in 2023 (8.12 per 100,000) remained higher than those in 2005. Spatially, incidence exhibited significant positive spatial correlation(Moran’s I: 0.097 in 2008 to 0.400 in 2010, all P-values < 0.001), with high-incidence counties concentrated in northern and central Xinjiang, while low-incidence counties were mainly located in the south. Northern Xinjiang, particularly areas centered around Tacheng Prefecture, Bortala Prefecture, and Ili Prefecture, was identified as a hotspot, whereas Kashgar and Hotan Prefectures in southern Xinjiang formed cold spots. Model analysis indicated that the STAR model provided the best fit(residual = 0.020 [−0.292, 0.317]，AIC=2,549.4), with year, spatial lag(β = 0.323), and temporal lag(β = 0.420) significantly affecting incidence rates (P < 0.05). The findings suggest that echinococcosis incidence in Xinjiang exhibits temporal continuity and spatial spillover effects.* ***Conclusion*** *: Echinococcosis in Xinjiang exhibited marked spatiotemporal heterogeneity during 2005–2023. While national and regional prevention programs contributed to reducing incidence after 2017, the burden remains substantial. These findings underscore the need for sustained and regionally coordinated prevention and control strategies to prevent potential resurgence.”*

Q7:

Lines 71-73: Change “The incidence data of echinococcosis reported from 2005 to 2023 in Xinjiang and 96 districts/counties were obtained from the China Infectious Disease Surveillance and Reporting System” to “Data on the incidence of echinococcosis reported in Xinjiang Uygur Autonomous Region and its 96 districts/counties during 2005–2023 were obtained from the China Infectious Disease Surveillance and Reporting System (IDSR).”

**Response：**Thank you for your valuable suggestion. We have revised the description of the data source by incorporating your comments and those of other reviewers to make it more accurate and precise:

Lines:99-110

*“Data on cases of echinococcosis in Xinjiang Uygur Autonomous Region and its 96 districts/counties during 2005–2023 were obtained from the China Infectious Disease Surveillance and Reporting System (IDSR), a national platform for mandatory infectious disease reporting. Cases were diagnosed according to the National Diagnostic Standard for Echinococcosis (WS 257-2006), which considers epidemiological history, clinical symptoms, imaging findings, and laboratory results. All reported cases were reviewed through multi-level verification at county, prefecture, regional, and national Centers for Disease Control and Prevention (CDC). The incidence data were directly obtained from IDSR and were not independently calculated by the authors. Because echinococcosis is a chronic disease, the reported incidence reflects the number of confirmed and reported cases within a given period rather than the true rate of new infections. ”*

Results/Discussion

Q8:

The authors indicate that the control program was initiated in 2005 with an incidence of 1.95/100,000, and then the incidence gradually increased to 10.8/100,000. The authors should discuss the reasons for this increase. Was the control program ineffective? Or were there other reasons for the rise? After 2017, the incidence decreased and remained at 3.99/100,000 in 2023, which is still higher than when the control program began. I have not seen a discussion of these results.

Response：Thank you very much for your valuable comments. In response to your concerns regarding the changes in reported incidence since 2005, we have supplemented and refined the Discussion section in the revised manuscript, providing a detailed description of the dynamic changes in reported incidence in relation to the implementation of control measures and their potential influencing factors. These revisions allow readers to better understand the long-term incidence trends and their relationship with relevant interventions.

Q9:

Line 298-300: “The subsidy for cystic echinococcosis has increased to 10,000, while the subsidy for alveolar echinococcosis has reached 20,000.” Suggestion: Specify the currency to avoid ambiguity.

Response：Thank you for pointing out the issue. We have clarified that all subsidy amounts are in Chinese Yuan (CNY).

Lines:344-346

*“...promoting the construction of comprehensive intervention zones, and raising special subsidy standards (10,000 Chinese Yuan (CNY) for cystic echinococcosis and 20,000 CNY for alveolar echinococcosis).”*

Reviewer #3:Edmundo Larrieu

Q1:It's always valuable to have new information from one of the most endemic areas in the world.

In the background, I suggest not mentioning mortality in sheep and goats. It's uncertain, and animals are usually slaughtered before the cysts severely damage their health. CE is a public health problem, and this issue should be emphasized. In livestock, it could only be of interest if there are CE confiscations in slaughterhouses. There is data!

**Response：**We thank the reviewer for this important suggestion. We agree that claims about mortality in sheep and goats are uncertain and can be misleading. We have removed the sentence referring to livestock mortality.

Another issue is that a long period of time is taken, but there's no mention of whether there are official control programs or what they consist of. According to the last paragraph of background, there is no program, and I don't believe that's the case. Please explain this, as it will be vital to interpreting the data found.

**Response：**Thank you very much for raising this important point. We fully agree that clearly describing official control programs and their content is essential for interpreting long-term trends in incidence. In response to your comments, we have made the following revisions in the updated manuscript:

In the Background section, we added a concise paragraph summarizing the major national and Xinjiang-level control programs since 2005 and their core measures, with relevant citations;

In Figure 1, we included a timeline that overlays the initiation or expansion of major programs on the annual reported incidence curve, allowing readers to more intuitively understand the relationship between policy measures and fluctuations in incidence;

In the Discussion section, we further elaborated on how reported incidence dynamically changed in relation to the implementation of these policies, clarifying the connection between policy actions and incidence trends.

With these revisions, we believe readers can better understand the policy context and interventions underlying long-term trends in reported echinococcosis incidence.

Lines: 76-82

*“To address this high prevalence, the Chinese government and Xinjiang regional authorities have implemented a series of echinococcosis control programs since 2005. These measures include free treatment for patients, regular dog deworming campaigns, targeted population screening, health education initiatives, and improvements in pastoral sanitation. Such programs have been crucial in enhancing case detection and reducing transmission in endemic areas [8-9].”*

Lines:325-366

*“Since December 2004, echinococcosis has been included in the management of Category C notifiable infectious diseases under the Infectious Disease Prevention and Control Law, and in 2005 the national control program was launched, with central government funds transferred to support control activities in Xinjiang [13-14]. In 2006, echinococcosis was designated as a major infectious disease eligible for free treatment. In 2010, the “Action Plan for Echinococcosis Control (2010–2015)” was implemented, with core measures including dog registration and regular deworming, slaughterhouse inspection and safe disposal, targeted population screening and treatment, improvement of drinking water in pastoral areas, and multi-level health education [15-17]. Since 2016, Xinjiang has launched a universal health check-up program, offering annual free examinations for registered residents, which substantially improved the capacity for early case detection [18]. Therefore, the continued increase in reported incidence from 2005 to 2017 reflects not only the cumulative effect of ongoing transmission but also a significant rise in case detection driven by policy initiatives and active screening.*

*From 2017 to 2023, the reported incidence of echinococcosis in Xinjiang declined significantly, with an APC of −17.71% (95% CI: −27.81 to −10.48), indicating the positive impact of control measures [19]. During this period, the government continued to increase financial and technical input, achieving routine surveillance in all 81 endemic counties by 2019, promoting the construction of comprehensive intervention zones, and raising special subsidy standards (10,000 Chinese Yuan (CNY) for cystic echinococcosis and 20,000 CNY for alveolar echinococcosis). In addition, extensive health education campaigns were implemented, effectively improving residents’ awareness and knowledge of prevention and control [20-21]. By 2019, prior to the COVID-19 pandemic, the reported incidence had fallen to 6.89 per 100,000, representing a 36.6% decrease from the 2017 peak. However, during the COVID-19 outbreak from 2020 to 2022, substantial prevention and control resources were redirected to pandemic response, limiting human and material inputs for echinococcosis control. Routine surveillance and intervention schedules were disrupted, with reduced monitoring frequency and delayed implementation of control measures in some areas, thereby weakening the effectiveness of interventions [22]. In this period, the reported incidence was 4.04, 3.65, and 3.11 per 100,000, respectively, showing a continued decline, though the decrease may partly reflect underreporting due to limited surveillance during the pandemic. In the post-pandemic era, close attention should be paid to the potential rebound of incidence. Ai J. and colleagues, using a SARIMA model, predicted a slight increase in incidence between 2022 and 2025 [23]. Our study found that in 2023, the reported incidence rebounded to 3.99 per 100,000, representing a 28.3% year-on-year increase and returning to the 2020 level. This single-year increase should be interpreted with caution, as it may partly reflect surveillance artifacts rather than a true resurgence. Nonetheless, the finding underscores the need to sustain early screening, health education, and multi-level interventions in the post-pandemic period.”*

The statistical analysis is avoiding the use of extensive information and speaks well of the data analysis.

Regarding the results, I personally think it's better to present the exact P values.

**Response：**Thank you for your valuable comment. Regarding the presentation of P values, the Joinpoint model we used applies the Empirical Quantile method, which does not provide exact P values. For P values smaller than 0.001, we report them as <0.001; for multiple results, we consistently report them as <0.05; all other results are presented with exact P values. In addition, we have further supplemented the data throughout the manuscript to ensure the analyses are comprehensive and detailed.

Again, the lack of information on programs and actions makes it difficult to know if the fluctuations are real. This is a problem with reporting, active case-finding campaigns, or the result of deworming dogs or vaccinating sheep.

Discussion. Finally, program actions and case-finding for asymptomatic cases are described. Therefore, the trends are clearly influenced by this.

**Response：**We sincerely thank the reviewer for the valuable comments. Based on your suggestions, we have made corresponding revisions and additions to the Discussion section. A timeline has been added in Figure 1, clearly marking the key control programs and intervention points, allowing a more intuitive correspondence with the annual reported incidence changes. In the Discussion, we have expanded the analysis of possible causes of temporal fluctuations, providing a more detailed account of the dynamic relationship between policy implementation and changes in reported incidence. In the absence of quantitative intervention data, we have qualitatively supported these explanations using the timeline and relevant literature, and we have explicitly acknowledged surveillance bias as a study limitation.

Furthermore, we suggest that future studies could conduct county-level comparative analyses (active case-finding vs. non-active case-finding) to more systematically evaluate the real effects of interventions. Through these revisions, we believe the Discussion now presents a clearer and more comprehensive explanation of the relationship between control measures and changes in reported incidence, and aligns more closely with our study findings.

Lines: 410-426

*“This study has several limitations. First, the early monitoring system in Xinjiang was incomplete, resulting in missing incidence data for certain years and counties, which affects the continuity of trend analysis and the completeness of spatial distribution research. Second, the reported incidence data reflect only diagnosed and registered cases, which may differ from the true infection rate due to underreporting or variations in surveillance coverage and active case-finding intensity. Third, the study lacks quantitative data on the implementation of control interventions (e.g., dog deworming, livestock vaccination, and active screening campaigns), which limits the ability to directly evaluate the impact of these measures on incidence trends. Fourth, this study mainly considers temporal and spatial factors, without incorporating climate change, socio-economic factors, and other potential determinants, which may limit a comprehensive understanding of the drivers of incidence changes.*

*Given these limitations, future research could incorporate climate and socio-economic variables, obtain quantitative intervention data [30], and conduct county-level comparative studies (e.g., active screening vs. non-active screening areas) to more systematically assess the effectiveness of control measures and further elucidate the epidemiological patterns of echinococcosis in Xinjiang.”*

In the graphs and throughout the work, avoid using hydatidosis and replace it with cystic echinococcosis.

**Response：**Thank you for this terminological clarification. We have replaced all uses of “hydatidosis” with “echinococcosis” throughout the text, figures, and tables.

However, it's already too late.

We suggest reorganizing the work, perhaps describing the control actions in the introduction. And, possibly, a timeline graphing the incidence by year and identifying the control or search actions initiated.

Clearly, the objective is poorly stated, as it proposes to advise authorities who are already working. It would be appropriate to change it to something like helping authorities evaluate the progress of actions. And these actions should be analyzed in the discussion.

**Response：**We sincerely thank the reviewer for their careful reading of our manuscript and valuable suggestions. Following your advice, we have added a timeline figure (Figure 1) showing the annual incidence and major control and screening activities from 2005 to 2023, which allows readers to better understand the temporal relationship between interventions and epidemiological changes.

Regarding the presentation of policy measures, we chose to retain them in the Discussion rather than moving them to the Introduction. This enables us to directly link control actions with study results—such as annual percentage change (APC), year-to-year incidence variation, and spatial distribution—providing a more coherent interpretation. In addition, we have substantially revised the Discussion to provide a clearer and more comprehensive analysis, highlighting the relationship between control measures and changes in incidence over time.

We hope these revisions address your concerns and make the presentation of control measures and their impact on reported incidence clearer and easier to interpret.

Lines325-366

*“Since December 2004, echinococcosis has been included in the management of Category C notifiable infectious diseases under the Infectious Disease Prevention and Control Law, and in 2005 the national control program was launched, with central government funds transferred to support control activities in Xinjiang [13-14]. In 2006, echinococcosis was designated as a major infectious disease eligible for free treatment. In 2010, the “Action Plan for Echinococcosis Control (2010–2015)” was implemented, with core measures including dog registration and regular deworming, slaughterhouse inspection and safe disposal, targeted population screening and treatment, improvement of drinking water in pastoral areas, and multi-level health education [15-17]. Since 2016, Xinjiang has launched a universal health check-up program, offering annual free examinations for registered residents, which substantially improved the capacity for early case detection [18]. Therefore, the continued increase in reported incidence from 2005 to 2017 reflects not only the cumulative effect of ongoing transmission but also a significant rise in case detection driven by policy initiatives and active screening.*

*From 2017 to 2023, the reported incidence of echinococcosis in Xinjiang declined significantly, with an APC of −17.71% (95% CI: −27.81 to −10.48), indicating the positive impact of control measures [19]. During this period, the government continued to increase financial and technical input, achieving routine surveillance in all 81 endemic counties by 2019, promoting the construction of comprehensive intervention zones, and raising special subsidy standards (10,000 Chinese Yuan (CNY) for cystic echinococcosis and 20,000 CNY for alveolar echinococcosis). In addition, extensive health education campaigns were implemented, effectively improving residents’ awareness and knowledge of prevention and control [20-21]. By 2019, prior to the COVID-19 pandemic, the reported incidence had fallen to 6.89 per 100,000, representing a 36.6% decrease from the 2017 peak. However, during the COVID-19 outbreak from 2020 to 2022, substantial prevention and control resources were redirected to pandemic response, limiting human and material inputs for echinococcosis control. Routine surveillance and intervention schedules were disrupted, with reduced monitoring frequency and delayed implementation of control measures in some areas, thereby weakening the effectiveness of interventions [22]. In this period, the reported incidence was 4.04, 3.65, and 3.11 per 100,000, respectively, showing a continued decline, though the decrease may partly reflect underreporting due to limited surveillance during the pandemic. In the post-pandemic era, close attention should be paid to the potential rebound of incidence. Ai J. and colleagues, using a SARIMA model, predicted a slight increase in incidence between 2022 and 2025 [23]. Our study found that in 2023, the reported incidence rebounded to 3.99 per 100,000, representing a 28.3% year-on-year increase and returning to the 2020 level. This single-year increase should be interpreted with caution, as it may partly reflect surveillance artifacts rather than a true resurgence. Nonetheless, the finding underscores the need to sustain early screening, health education, and multi-level interventions in the post-pandemic period.”*
