# Supplementary material for: Spatiotemporal distribution and control of echinococcosis in Xinjiang, 2005–2023
Source: PLoS Negl Trop Dis. 2025 Dec 19;19(12):e0013775. doi: 10.1371/journal.pntd.0013775 (PMC12716753; doi:10.1371/journal.pntd.0013775)
Supplement: Attachment — Submitted filename: Response to Reviewers11.17.docx [file pntd.0013775.s005.docx]

PNTD-D-25-00532
Spatiotemporal Distribution and Control of Echinococcosis in Xinjiang, 2005-2023
PLOS Neglected Tropical Diseases

**Reviewer #2:** still some spelling errors need to be carefully checked and changed, such as "Echinococcusis or echinococcusis" should be "Echinococcosis or echinococcosis". I wish to accept for publication.

**Response：**Thank you for your careful review. We have thoroughly checked the entire manuscript and corrected all instances of the misspelled "Echinococcusis"

**Reviewer #3:**

Q1

I believe it would help better interpret the work if the objectives of the study were clearly stated before the methods.

**Response：**We have clarified the study objectives in the Introduction to provide a clear context before the Methods section, facilitating better interpretation of the work.

**Lines:90-99**

*“Given the potential public health threat posed by echinococcosis in Xinjiang, a comprehensive understanding of its epidemiological dynamics is essential for improving prevention and control efforts. In this context, the present study systematically analyzed the spatiotemporal distribution of echinococcosis in Xinjiang from 2005 to 2023, aiming to characterize temporal trends and spatial clustering at the county level, summarize the implementation of major control measures during the study period, and evaluate the temporal and spatial associations between comprehensive control activities and disease incidence. The findings provide an evidence-based foundation for refining control strategies and optimizing public health resource allocation in endemic regions.”*

Q2
Another issue, necessary for subsequent data analysis, is a summary presentation of the control strategies applied during the study period. For example, are dogs regularly dewormed? Are sheep vaccinated? Since when? Are there massive active search programs using ultrasound? Do these activities cover the entire region or are they limited to certain areas?

**Response：**In accordance

**Lines:122-149**

*“Overview of Echinococcosis Control and Surveillance*

*Echinococcosis in Xinjiang predominantly occurs in 81 counties (cities, districts). Since 2006, systematic control efforts have been implemented in endemic areas, focusing on comprehensive strategies including the control of infection sources, intermediate host management, population screening and treatment, and health education. By 2016, control measures had been implemented across all echinococcosis-endemic counties.*

*For human population protection, targeted groups in endemic areas—such as herders, slaughterhouse workers, and children—underwent regular ultrasound screening, and confirmed cases were promptly enrolled in standardized treatment programs. Health education was conducted through informational brochures, village-level lectures, and bilingual videos to disseminate knowledge on prevention and enhance residents’ self-protection awareness.For animal control, dogs were primarily subjected to a monthly deworming program, with excreta from treated animals handled safely and hygienically. Livestock control relied mainly on echinococcosis vaccination, with newborn lambs receiving two rounds of immunization to reduce intermediate host infection rates.*

*Regarding surveillance, from 2016 to 2018, echinococcosis monitoring was conducted in 39 counties. Beginning in 2019, the monitoring scope was expanded to cover all 81 endemic counties. In addition, since 2019, pilot comprehensive intervention programs have been implemented in three counties to explore more effective control strategies. It should be noted that all medical institutions across Xinjiang are required to report newly diagnosed cases in accordance with national regulations for Class C infectious diseases, regardless of whether the area is covered by control or surveillance programs. This integrated system of control and surveillance, which covers key hosts and high-risk populations and combines health education with case management, has provided systematic support for the reduction of echinococcosis incidence.”*

Q3
Method: Does the surveillance system distinguish new cases from reoperations or readmissions? Is the incidence calculated by excluding reoperations or readmissions?

**Response：**In accordance with national reporting requirements, only newly diagnosed cases are reported by medical institutions, and reoperations or readmissions of previously confirmed cases are excluded. All reports submitted to IDSR undergo verification by local CDC offices to ensure completeness and to identify and remove any duplicate or erroneous entries. Consequently, the incidence reported in our study reflects only newly diagnosed and reported cases.

**Lines:108-110**

*“ Only newly diagnosed cases were reported, and all case reports were verified through multi-level review by county, prefecture, regional, and national Centers for Disease Control and Prevention (CDC) to ensure accuracy and eliminate duplication. ”*

Q4
Discussion: The association with control appears here, but it is confusing. Please describe the most important aspects as suggested in the introduction, and the discussion should clarify the impact of these actions on the trend.

**Response：**In accordance

**Lines:358-385**

*“Since 2005, echinococcosis has been included in the management of Category C notifiable infectious diseases under the Infectious Disease Prevention and Control Law, and in 2005 the national control program was launched, with central government funds transferred to support control activities in Xinjiang [13-14]. In 2006, echinococcosis was designated as a major infectious disease eligible for free treatment. In 2010, the “Action Plan for Echinococcosis Control (2010–2015)” was implemented, with core measures including dog registration and regular deworming, slaughterhouse inspection and safe disposal, targeted population screening and treatment, improvement of drinking water in pastoral areas, and multi-level health education [15-17]. By 2016, comprehensive control measures had been implemented all 81 endemic counties, and Xinjiang had also launched a universal health check-up program, providing annual free examinations for registered residents, which substantially improved the capacity for early case detection [18]. Therefore, from 2005 to 2017, the reported incidence increased, peaking in 2017. This rise reflected both ongoing transmission and intensified case detection driven by population-based screening, particularly among high-risk groups.*

*From 2017 to 2023, the reported incidence of echinococcosis in Xinjiang declined significantly, with an APC of −17.71% (95% CI: −27.81 to −10.48), indicating the positive impact of control measures [19]. During this period, the government continued to strengthen financial and technical support, achieving routine surveillance in all 81 endemic counties by 2019, promoting the construction of comprehensive intervention zones, maintaining dog management and livestock vaccination strategies, sustaining continuous health education, and implementing active population screening, all of which contributed to reducing transmission. In addition, the government raised special subsidy standards (10,000 Chinese Yuan (CNY) for cystic echinococcosis and 20,000 CNY for alveolar echinococcosis)[20-21]. By 2019, prior to the COVID-19 pandemic, the reported incidence had fallen to 6.89 per 100,000, representing a 36.6% decrease from the 2017 peak. ”*
